# From Mistreatment to Burnout: The Mediating Role of Emotional Regulation in Graduate Medical Trainees

**DOI:** 10.5334/pme.2126

**Published:** 2025-10-21

**Authors:** Binbin Zheng, Jerusalem Merkebu, Ting Dong, Jerri Curtis, TingLan Ma, Steven J. Durning, Michael Soh

**Affiliations:** 1 Uniformed Services University of the Health Sciences, US; 2 University of Maryland, Baltimore, US

## Abstract

**Purpose:**

Mistreatment in medical training environments has been linked to emotional exhaustion, yet the underlying mechanisms remain underexplored. This study examines the mediating role of emotional regulation and social support in the relationship between mistreatment and burnout among graduate medical trainees.

**Method:**

We conducted a cross-sectional survey of 266 graduate medical trainees from one of the largest GME institutions in the northeastern United State. The survey included measures of workplace mistreatment, emotional regulation, social support, and burnout. Path analysis was employed to test direct and indirect relationships among mistreatment, emotional regulation, social support, and burnout.

**Results:**

Mistreatment was significantly associated with burnout, with personal attacks demonstrating a strong direct effect (β = 0.36) and a substantial total effect (β = 0.55). Emotional dysregulation partially mediated this relationship, with a significant indirect effect (β = 0.16). Suppressive emotional regulation strategies also contributed to burnout. Social support played a protective role, reducing the impact of mistreatment on burnout.

**Conclusions:**

These findings underscore the detrimental impact of mistreatment on medical trainee well-being and the critical role of emotional regulation and social support in mitigating burnout. Institutional interventions should focus on strengthening peer and leadership support networks, and integrating emotional regulation training into residency programs. Addressing these factors can enhance resilience, improve professional fulfillment, and promote a more supportive medical training environment.

## Introduction

Workplace mistreatment and burnout among graduate medical trainees remain significant concerns in graduate medical education, with significant implications for both individual well-being and patient care outcomes [1,2,3]. Mistreatment, which includes behaviors such as public humiliation, discrimination, and intimidation, is alarmingly common and can create a toxic learning environment. A recent study indicates that 39% of residents report experiencing at least one incident during their training period, most commonly in the form of public humiliation [2]. These negative experiences often elicit intense emotional responses, such as anger, frustration, and fear, which, if poorly managed, can contribute to emotional exhaustion and ultimately lead to burnout [4,5].

Emotional regulation, which is defined as the process of identifying, managing, and modulating emotional responses to stressors, plays a crucial role in determining how individuals cope with these experiences [6,7]. Roth et al. [8] proposed three key dimensions of emotional regulation: integrative regulation, emotional dysregulation, and suppressive regulation. Integrative regulation reflects the ability to process and utilize emotional experiences constructively, prompting adaptive responses to stressors. Emotional dysregulation, on the other hand, captures difficulties in managing and modulating emotions, often resulting in heightened emotional reactivity or poor coping. Suppressive regulation refers to the tendency to inhibit outward emotional expression, which can provide short-term relief but often leads to long-term psychological strain.

Additionally, Gross’s Process Model of Emotion Regulation [6] highlights intrinsic and extrinsic aspects of regulation strategies, distinguishing between antecedent-focused approaches, such as situation selection and cognitive appraisal, and response-focused approaches, like suppression. Social support also plays a crucial role in amplifying the effectiveness of extrinsic strategies. Without external encouragement and intrinsic regulation capacities, individuals may resort to suppressing their emotional responses, leading to increased psychological strain over time. Thus, by examining the intrinsic and extrinsic dimensions of the emotional regulation model, we see how proactive individual behaviors and social relationships with peers, mentors, family, and friends can promote adaptive regulation strategies.

The aforementioned emotional regulation strategies are important as *burnout*, characterized by emotional exhaustion, depersonalization, and a reduced sense of personal accomplishment, has been linked to decreased professional satisfaction, lower quality of patient care, and higher attrition rates in medical training programs [9,10]. Addressing factors that exacerbate burnout, including mistreatment, is therefore critical for fostering healthier and more supportive training environments.

Although the relationship between mistreatment and burnout is well-established, the critical role of emotional regulation strategies and social support in mediating this connection remains underexplored, especially among graduate medical education (GME) trainees. Understanding how GME trainees regulate their emotional responses to mistreatment is crucial for identifying points of intervention. By targeting emotional regulation, it may be possible to develop evidence-based strategies that mitigate the negative consequences of mistreatment, enhance resilience, and promote a positive and productive training environment.

This study aims to address this gap by examining the interplay between mistreatment, emotional regulation, and burnout among medical residents. We propose that workplace mistreatment contributes to increased burnout among medical residents. However, this relationship is expected to be mediated by emotional regulation strategies and social support. Two research questions proposed in this study include:

1. How do different emotional regulation strategies mediate the relationship between workplace mistreatment and burnout among graduate medical trainees?
2. How does social support mediate the relationship between workplace mistreatment and burnout among graduate medical trainees?

## Methods

### Study site and participants

The participants of the study were from one of the largest GME sponsors in the northeast region of the U.S. At the time of the study (Spring 2024), it had a total of 62 programs and 675 interns/residents/fellows. All the residents and fellows were invited to participate in the study. This study was approved by the Institutional Review Board from the designated university (protocol number DBS.2023.596).

### Measures

#### Mistreatment

For workplace mistreatment, we used two scales – Workplace Psychologically Violent Behaviors (WPVB) [11] and the Negative Acts Questionnaire Revised (NAQ_R) scale [12]. The original WPVB scale measured workplace psychologically violent actions with four subscales (individual’s isolation from work, attack on professional status, attack on personality, and direct attack) and had been used with a population of nurses [13]. In our study, we selected one subscale that was relevant to the clinical learning environment: attack on professional status. A sample survey item is “how often have you experienced: always have your professional adequacy questioned in the work you do”. Seven items were included using a five-point Likert scale from “Never faced this” to “I constantly faced this”. The NAQ_R scale measured workplace bullying with the definition being “repeated actions and practices directed against one or more workers, that are unwanted by the victim, carried out deliberately or unconsciously, that cause humiliation, offense, and distress and can interfere with work performance and/or cause an unpleasant working environment.” The scale was used in a national assessment of workplace bullying of the U.S. surgeon population which included residents [14]. A sample survey item is “Within the last six months, how often at work have you had insulting or offensive remarks made about you”. Six items were included using a five-point Likert scale from “Not in the past six months” to “Daily or almost daily”.

#### Emotional regulation

Emotional regulation was measured using survey items adapted from the Emotional regulation scales [8]. This measure includes three key dimensions: emotion dysregulation (difficulties in managing and modulating emotional responses), suppressive regulation (attempting to inhibit outward expression of emotions), and integrative regulation (acknowledging, understanding, and constructively using emotions to guide decisions and behavior). Ten items were included using a five-point Likert scale.

#### Burnout

Burnout was assessed using two items — emotional exhaustion and depersonalization — from the Medical Student Well-being Index (MSWBI) [15], by changing “medical school” to “training program.” In addition, to increase the granularity of responses and align with the ACGME Resident/Fellow Survey, we used a five-point Likert scale (Strongly disagree, Disagree, Neither agree nor disagree, Agree, Strongly agree) instead of a binary option (Yes or No) as adopted in the original MSWBI.

#### Social support

We adopted the Brief Social Support Scale from Beutel [16] to measure social support. The scale includes four items using a four-point Likert scale. A sample item is “how often is someone available to allow you to vent your emotion in front of this person?”

### Survey distribution

The survey was anonymous and distributed via a generic link generated by Qualtrics. This link was sent to the email list of all eligible interns, residents, and fellows multiple times. The data collection period was from early December 2023 to late January 2024. There was no incentive for participating in the study.

### Data Analysis

We conducted a path analysis using lavaan 0.6.16 to test our hypothesized mediation model. Path analysis was chosen for its ability to simultaneously estimate multiple pathways and test complex mediational hypotheses while accounting for measurement error [17]. The analysis was conducted using maximum likelihood (ML) estimation with a sample of 160 participants (from a total of 266 cases).

Model fit was assessed using multiple indices following contemporary recommendations: Chi-square test, Comparative Fit Index (CFI), Tucker-Lewis Index (TLI), Root Mean Square Error of Approximation (RMSEA) with its 90% confidence interval, and Standardized Root Mean Square Residual (SRMR). While there is ongoing debate about cutoff values, generally accepted thresholds suggest good fit when CFI > .95, RMSEA < .06, and SRMR < .08 [18].

We tested a comprehensive mediation model examining both direct and indirect effects of workplace incivility (measured through WPVB subscales and NAQ) on well-being/burnout through the following mediating mechanisms: three emotional regulation (emotion dysregulation, suppressive coping, and integrative regulation) and social support. The significance of indirect effects was evaluated using standard errors and z-tests. The model included estimation of total effects, decomposing the relationship between workplace incivility and burnout into direct and indirect pathways through each mediator. Models were estimated with maximum likelihood with robust standard errors (MLR) to address non-normality, and we report robust (scaled) χ², CFI/TLI, RMSEA, and SRMR.

To assess model quality, we examined R-square values for endogenous variables and standardized path coefficients. Standardized estimates are reported to facilitate interpretation, with statistical significance evaluated at *p* < .05. The analysis accounted for all specified paths simultaneously, allowing us to control for relationships among mediators while estimating their unique effects on the outcome.

## Results

A total of 266 survey responses were received from 675 graduate medical trainees, yielding a response rate of 39.4%. This response rate is consistent with typical response rates reported in physicians and trainee survey research, which indicated a range of 20% and 40% being acceptable, given competing clinical demands, survey fatigue, and limited discretionary time [19,20]. Respondents represented a range of training levels, with 57.8% being senior trainees (PGY-3s and above) and 42.2% junior trainees (PGY-1s and 2s). The sample also included trainees from various specialties, with 8.4% in surgical specialties and 91.6% in non-surgical specialties. Male trainees comprised 67.5% of respondents, comparable to the overall institutional trainee population (64% male).

The descriptive statistics for all survey items and their correlations are presented in Table 1. The mean values indicate that burnout was moderately reported (*M* = 2.70, *SD* = 1.07). Mistreatment (*M* = 1.57, *SD* = 0.55) was significantly correlated with burnout (*r* = .56, *p* <.01). Of the two sub-scales of mistreatment, attack (*M* = 1.86, *SD* = 0.84) was more frequently reported than negative act (*M* = 1.12, *SD* = 0.30). Regarding emotional regulation strategies, integrative regulation was reported most frequently (*M* = 3.60, *SD* = 1.06), followed by suppressive regulation (*M* = 3.05, *SD* = 1.20) and dysregulation (*M* = 2.21, *SD* = 1.06). Both dysregulation and suppressive regulation showed positive associations with burnout (*r* = .56 and .33, respectively, *p* < .01). Social support (*M* = 3.46, *SD* = 0.75) was reported as above average and demonstrated a negative correlation with burnout (*r* = –.41, *p* < .01). The reliability analysis of the measured dimensions, assessed using Cronbach’s alpha, indicated strong internal consistency across most variables, ranging from 0.702 to 0.968, indicating that the instruments used were effective in capturing the construct under investigation.

**Table 1:** Means, standard deviations, and correlations with confidence intervals.


VARIABLE	*M*	*SD*	1	2	3	4	5	6

1. Burnout	2.66	1.08						

2. Attack	1.86	0.84	.58**					

			[.46, .67]					

3. Negative act	1.12	0.30	.41**	.57**				

			[.28, .53]	[.45, .67]				

4. Dysregulation	2.21	1.06	.56**	.47**	.34**			

			[.45, .66]	[.34, .58]	[.20, .47]			

5. Suppressive	3.05	1.20	.33**	.25**	.09	.30**		

			[.18, .46]	[.10, .39]	[–.06, .24]	[.15, .43]		

6. Integrative	3.60	1.06	.13	.14	.00	.20*	.16*	

			[–.03, .28]	[–.02, .28]	[–.15, .16]	[.04, .34]	[.00, .30]	

7. Social support	3.46	0.75	–.41**	–.36**	–.37**	–.34**	–.31**	.15

			[–.53, –.27]	[–.49, –.22]	[–.50, –.23]	[–.47, –.19]	[–.44, –.16]	[–.00, .30]


*Note. M* and *SD* are used to represent mean and standard deviation, respectively. Values in square brackets indicate the 95% confidence interval for each correlation. The confidence interval is a plausible range of population correlations that could have caused the sample correlation (Cumming, 2014). * indicates *p* < .05. ** indicates *p* < .01.

### Model modification

**Figure 1:**
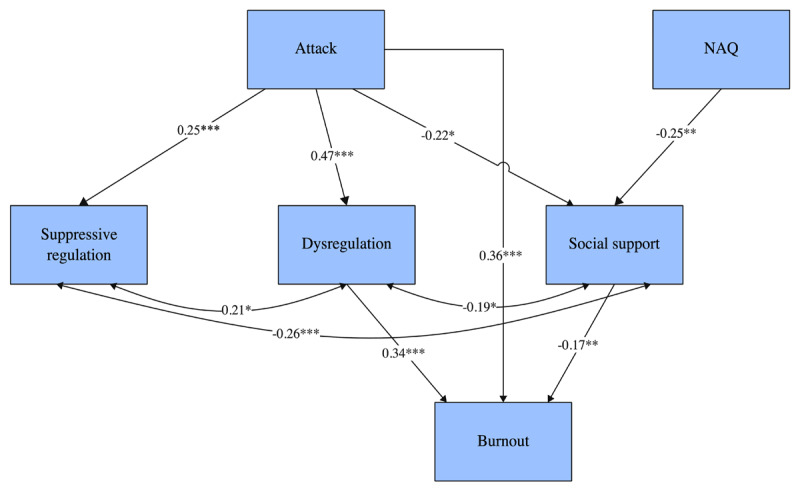
Results of the path analysis (coefficients are standardized).

Given the lack of bivariate association between integrative regulation and burnout, we excluded integrative regulation from the subsequent path analysis model. The mediation model therefore focused on the two emotional regulation strategies that demonstrated significant relationships with burnout: dysregulation and suppressive regulation. In addition, examination of parameter estimates revealed several non-significant pathways, including NAQ->burnout, NAQ->dysregulation, suppressive regulation->burnout, and NAQ->suppressive regulation. Following model modifications by removing non-significant pathways mentioned above, the revised model demonstrated good fit: scaled χ²(3) = 5.41, *p* = .144; CFI(robust) = .992; TLI(robust) = .961; RMSEA(robust) = .058 (90% CI [.00, .135]); SRMR = .024. All fit indices met conventional acceptability criteria (CFI/TLI ≥ .95, RMSEA ≤ .08, SRMR ≤ .05), indicating good model-data correspondence. The results of the path analysis (see Table 2 and Figure 1) are summarized below.

**Table 2:** Results of Path Analysis.


PREDICTOR	OUTCOME	ESTIMATE	Std. ERROR	z-value	p-value	Std. ESTIMATE

Attack	Burnout	0.464	0.085	5.457	< .001	0.359

Dysregulation	Burnout	0.349	0.071	4.876	< .001	0.34

Social Support	Burnout	–0.238	0.083	–2.882	0.004	–0.165

Attack	Dysregulation	0.588	0.089	6.623	< .001	0.466

Negative act	Social Support	–0.634	0.221	–2.863	0.004	–0.252

Attack	Social Support	–0.194	0.093	–2.077	0.038	–0.217

Attack	Suppressive	0.36	0.094	3.814	< .001	0.253


### Mistreatment and Burnout

Personal attacks had a significant direct effect on burnout (*β* = 0.36, *p* < 0.001). The total effect of personal attacks on burnout was substantial (*β* = 0.55, *p* < 0.001). In contrast, NAQ showed a weaker and non-significant direct relationship with burnout in the model, suggesting that more subtle forms of workplace mistreatment may primarily influence well-being through indirect pathways rather than direct effects. However, NAQ significantly impacted social support (*β* = –0.25, *p* < 0.01), indicating that these more pervasive workplace behaviors may undermine the social resources that protect against burnout.

### Emotional Regulation and Coping Mechanisms

Personal attacks significantly impacted emotion dysregulation (*β* = 0.47, *p* < 0.001) and suppressive regulation (*β* = 0.25, *p* < 0.01). The relationship between personal attacks and burnout was partially mediated by emotion dysregulation, with a small but significant indirect effect (*β* = 0.16, *p* < 0.001).

### Social Support and Professional Relationships

Both personal attacks and negative workplace acts were significantly negatively associated with social support (*β* = –0.22, *p* < 0.05 and *β* = –0.25, *p* < 0.01 respectively). Social support demonstrated a protective effect against burnout (*β* = –0.13, *p* < 0.05).

## Discussion

Our study provides insights into the complex dynamics between mistreatment, emotional regulation, and social support in predicting burnout among GME trainees. The findings highlight the intricate relationship between emotional dysregulation, suppression, and burnout, emphasizing the multifaceted nature of workplace stressors and the critical need for targeted interventions to mitigate these challenges.

### The prominent role of emotional regulation in coping with mistreatment

The strong direct relationship between personal attacks and burnout, as well as the substantial total effect, underscores the pervasive impact of mistreatment on trainee well-being. Consistent with prior research, mistreatment, such as public belittlement during rounds and discriminatory practices, exacerbates stress and diminishes professional satisfaction [21]. These experiences of hopelessness and disengagement align with the concept of learned helplessness [22], underscoring the necessity for institutional policies that foster a culture of respect, promote accountability, and provide avenues for reporting mistreatment without fear of retaliation. However, it is worth noting that while some aspects of mistreatment can – and do – have a negative impact on trainees, other perceptions of mistreatment such as being interrupted on rounds, being corrected on rounds or in patient notes, or being asked questions that one may not know the answers to, may be more nuanced and complicated to disentangle. These situations can blur the line between mistreatment and legitimate educational practices. Thus, navigating this ongoing tension and balance is critical in ensuring appropriate corrective and formative actions, e.g. trainee interventions, faculty development, institutional policies, etc., are taken.

Our study also emphasized the mediating role of emotional dysregulation in the relationship between mistreatment and burnout. Personal attacks significantly influenced emotion dysregulation and suppressive regulation, with a small but significant indirect effect on burnout. The modest size of this effect suggests that while dysregulation plays a role, the pathway from mistreatment to burnout is not fully explained by emotional regulation alone. Other mechanisms, such as erosion of professional identity and systemic culture pressures, likely account for additional variance in how mistreatment led to burnout. These findings align with Gross’s work on the detrimental effects of maladaptive strategies such as suppression. Trainees often adopt stoic professionalism in response to mistreatment, which, while culturally reinforced in medical settings, exacerbates emotional strain. Promoting adaptive emotional regulation strategies, such as cognitive appraisal and mindfulness, may enhance resilience and reduce burnout [7].

### Interaction between social support, burnout, and mistreatment

The protective role of social support in mitigating the adverse effects of mistreatment was evident in our study as supported by prior research [23,24]. While social support demonstrated a protective effect against burnout, the magnitude of this association was mild compared to the stronger effects of mistreatment and emotional dysregulation. This may reflect the limits of social support when systemic mistreatment persists. In addition, the significant negative impact of personal attacks and negative workplace acts on social support suggests that hostile work environments can undermine critical support systems. When trainees experience mistreatment, they may withdraw from social interactions, perceive colleagues as less trustworthy, or feel isolated from institutional networks meant to provide emotional and professional reinforcement [25,26]. This dynamic highlights how mistreatment not only has direct effects on burnout but also undermines protective social networks, creating a reinforcing cycle of isolation and vulnerability.

Consistent with Cohen and Wills’s [27] buffering hypothesis, our findings highlight the value of social support, such as peer networks, supportive supervisors, and family relationships, in alleviating stress and supporting opportunities for extrinsic regulation. Social support acts as a mediator by reducing the emotional toll of stressors and providing alternative perspectives, encouragement, and guidance in difficult professional situations [28]. Prior research has demonstrated that individuals with strong social support experience lower job-related stress and greater resilience against burnout [9], particularly in high-stress environments such as medical training, where mentorship and collegiality play crucial roles in professional development and psychological well-being [29].

Interventions aimed at fostering social support within medical training environments are crucial for mitigating the negative consequences of mistreatment. Peer mentorship programs have been shown to enhance belonging, provide guidance on navigating professional challenges, and reinforce positive coping mechanisms [30,31]. Similarly, supervisor training can cultivate a culture of psychological safety by equipping leaders with the skills to recognize and address mistreatment while fostering open communication and mutual respect [32]. Implementing structured team-based interventions, such as workplace affinity groups or reflective practice sessions, may also help medical trainees feel more supported and valued within their institutions [33]. Whether through interpersonal interactions, leadership training, or teamwork-based initiatives, fostering connectedness, belonging, respect, and self-awareness during training may encourage adaptive emotional regulation strategies.

Despite the protective effects of social support, our findings indicate that negative workplace experiences can weaken support systems, exacerbating stress and burnout. Institutional efforts to address mistreatment should not only focus on preventing negative behaviors but also on actively strengthening social networks that provide essential psychological and professional reinforcement. Thus, a holistic approach that integrates intrinsic and extrinsic regulation interventions, mentorship programs, and leadership training is essential for fostering resilient and thriving medical workforce.

### Implications and future directions

The interplay between mistreatment, emotional regulation, and social support suggests a multi-pronged approach to intervention. First, addressing the root causes of mistreatment through institutional policy and cultural change is essential. In addition to policy, effective strategies may include faculty development, peer-led feedback workshops, structured debriefing, and leadership training that emphasizes respect, accountability, and psychological safety. Second, integrating emotional regulation training into residency programs can equip trainees with tools to manage stress more effectively. Such training could include guided reflection, mindfulness-based approaches, and wellness curricula designed to build emotional awareness and self-regulatory capacity. Third, promoting robust social support networks within training environments can serve as a protective buffer against the detrimental effects of workplace stressors. Programs that cultivate mentorship, peer connection, and collegiality may foster a greater sense of belonging. A summary of recommendations for addressing mistreatment in GME is provided in Table 3.

**Table 3:** Recommendations for addressing mistreatment in GME.


DOMAIN	RECOMMENDATIONS	EXAMPLES/STRATEGIES

Institutional culture	Move beyond policy to foster respect, accountability, and transparency	Develop policies in collaboration with residents; ensure confidentiality and transparent follow-up; commit to sustained culture change and accountability

Reporting & accountability	Strengthen trust in reporting mechanisms and ensure consequences for mistreatment	Establish robust, anonymous reporting systems; conduct regular climate assessments; mandate transparent follow-up by program directors

Faculty & leadership development	Create psychological safety; Improve feedback delivery and recognition of mistreatment	Leadership training in accountability and psychological safety; coaching on effective feedback delivery; integrate culture metrics into faculty evaluation

Emotional regulation	Support adaptive coping strategies	Mindfulness training, cognitive reappraisal skills, wellness curricula; provide protected time for reflection; normalize counseling and therapy access

Social support & mentorship	Build strong, reliable support networks within and across training levels	Implement structured peer mentorship; support affinity groups

System-level reform	Coordinate efforts across institutions to ensure sustainability and inclusion	Align institutional, hospital, and accreditation requirements; mandate long-term monitoring of outcome.


Additionally, while no correlation was found between integrative regulation and burnout, further examination of Roth’s emotional regulation scale suggests that integrative regulation often involves metacognitive reflection—a skill that may be challenging for medical trainees [34,35]. Building on this foundation, future research could draw on Gross’s process model of emotional regulation to further explore how interpersonal and contextual factors, such as relationships with peers, mentors, family, and friends, promote adaptive regulation strategies in medical trainees.

Longitudinal relationships between these factors and the effectiveness of targeted interventions should be further investigated. Besides, examining how cultural norms within medical training environments shape emotional regulation and support dynamics could provide valuable insights for tailoring interventions. Lastly, determining how different emotional regulation frameworks apply to various phases of medical education may yield more robust, context-specific data for medical trainees.

We would also like to acknowledge that the conceptual boundaries between mistreatment and unprofessional behavior remain ambiguous. Not all harmful encounters are formally categorized as mistreatment, and some behaviors may be simultaneously professional and psychologically harmful. As Gan and Snell [36] have argued, what constitutes mistreatment may vary considerably across programs and cultural contexts, and even among learners within the same context. Subtle yet harmful behaviors, such as microaggressions, exclusion from learning opportunities, or lack of psychological safety, are frequently overlooked in existing assessments. Adding to this complexity, perceptions of certain encounters, such as being corrected in front of peers, can differ widely. A constructive critique intended to support learning may feel supportive to one trainee but humiliating to another, particularly in high-stakes, public settings. This subjectivity underscores the importance of distinguishing between necessary corrective feedback and behaviors that cross into mistreatment, while also recognizing that learners’ experiences and perceptions are a critical part of the equation. Therefore, assessment tools should strive to capture both objective indicators of unprofessional conduct and the subjective impact of behaviors, ensuring that the nuance between constructive feedback, cultural norms, and genuinely harmful interactions is preserved. In addition, further qualitative studies are needed to examine these nuanced dynamics in depth, capturing how trainees and faculty interpret and experience potentially harmful encounters across clinical and cultural contexts.

### Limitations

Our study had a 39.4% survey response rate. Potential non-response and sampling bias may exist, as residents from psychiatric specialties might be more metacognitively aware of their emotions and emotional regulation strategies, making them more inclined to participate. Second, while our institution does have an anonymous mistreatment reporting system, we were not able to compare our data with existing official reports of mistreatment at our site, which may have allowed for some verification of our WPVB and NAQ findings. Finally, while both WPVB and NAQ demonstrate strong psychometric properties in general healthcare populations, they may not capture all of the complexity and nuance of medical residency training. We would call for future studies to develop a more tailored and resident-focused mistreatment instrument that could capture both the subtle forms of mistreatment under GME’s unique cultural and hierarchical dynamics.

## Conclusion

This study highlights the important impact of mistreatment on burnout, with emotional regulation serving as a key mediator and social support playing a critical protective role. By addressing these interconnected factors, medical training programs can foster healthier, more supportive environments that promote trainee well-being and professional fulfillment. Implementing targeted emotional regulation interventions, mentorship initiatives, and institutional policies aimed at reducing mistreatment, are essential to fostering resilience and sustaining a positive learning environment for future medical professionals.

## Disclaimer

This work was prepared by civilian employees of the US Government as part of the individuals’ official duties and therefore is in the public domain. The opinions and assertions expressed herein are those of the authors and do not necessarily reflect the official policy or position of the Uniformed Services University or the Department of Defense.

## References

[1] Dyrbye LN, West CP, Satele D, et al. Burnout Among U.S. Medical Students, Residents, and Early Career Physicians Relative to the General U.S. Population. Acad Med. 2014;89(3):443–451. DOI: 10.1097/ACM.0000000000000134 24448053

[2] Hammoud MM, Appelbaum NP, Wallach PM, et al. Incidence of Resident Mistreatment in the Learning Environment Across Three Institutions. Med Teach. 2021;43(3):334–340. DOI: 10.1080/0142159X.2020.1845306 33222573

[3] Hu YY, Ellis RJ, Hewitt DB, et al. Discrimination, Abuse, Harassment, and Burnout in Surgical Residency Training. N Engl J Med. 2019;381(18):1741–1752. DOI: 10.1056/NEJMsa1903759 31657887 PMC6907686

[4] Cheng MY, Neves SL, Rainwater J, et al. Exploration of Mistreatment and Burnout Among Resident Physicians: a Cross-Specialty Observational Study. Med Sci Educ. 2020;30(1):315–321. DOI: 10.1007/s40670-019-00905-z 34457673 PMC8368104

[5] Yau BN, Chen AS, Montgomery KB, Dubuque N, McDowelle DM. An Internal Perspective: the Psychological Impact of Mistreatment. Acad Psychiatry. 2021;45(3):308–314. DOI: 10.1007/s40596-021-01430-1 33709287

[6] Gross JJ. The emerging field of emotion regulation: An integrative review. Rev Gen Psychol. 1998;2(3):271–299. DOI: 10.1037/1089-2680.2.3.271

[7] Peña-Sarrionandia A, Mikolajczak M, Gross JJ. Integrating emotion regulation and emotional intelligence traditions: a meta-analysis. Front Psychol. 2015;6:160. DOI: 10.3389/fpsyg.2015.00160 25759676 PMC4338658

[8] Roth G, Vansteenkiste M, Ryan RM. Integrative emotion regulation: Process and development from a self-determination theory perspective. Dev Psychopathol. 2019;31(3):945–956. DOI: 10.1017/S0954579419000403 31113502

[9] Shanafelt TDMD, Hasan OMMPH, Dyrbye LNMDM, et al. Changes in Burnout and Satisfaction With Work-Life Balance in Physicians and the General US Working Population Between 2011 and 2014. Mayo Clin Proc. 2015;90(12):1600–1613. DOI: 10.1016/j.mayocp.2015.08.023 26653297

[10] Maslach C, Leiter MP. Understanding the burnout experience: recent research and its implications for psychiatry. World Psychiatry. 2016;15(2):103–111. DOI: 10.1002/wps.20311 27265691 PMC4911781

[11] Dilek Y, Aytolan Y. Development and psychometric evaluation of workplace psychologically violent behaviours instrument. J Clin Nurs. 2008;17(10):1361–1370. DOI: 10.1111/j.1365-2702.2007.02262.x 18416783

[12] El Ghaziri M, Storr CL, Simons SR, et al. Comparative psychometric review of the Negative Acts Questionnaire-Revised in a unionized U.S. public sector workforce. Work J Prev Assess Rehabil. 2019;62(1):161–171. DOI: 10.3233/WOR-182851 30689599

[13] Rutherford A, Rissel C. A survey of workplace bullying in a health sector organisation. Aust Health Rev Publ Aust Hosp Assoc. 2004;28(1):65–72. DOI: 10.1071/AH040065 15525252

[14] Pei KY, Hafler J, Alseidi A, Slade MD, Klingensmith M, Cochran A. National Assessment of Workplace Bullying Among Academic Surgeons in the US. JAMA Surg. 2020;155(6):524–526. DOI: 10.1001/jamasurg.2020.0263 32236505 PMC7113829

[15] Dyrbye LN, Szydlo DW, Downing SM, Sloan JA, Shanafelt TD. Development and preliminary psychometric properties of a well-being index for medical students. BMC Med Educ. 2010;10(1):8. DOI: 10.1186/1472-6920-10-8 20105312 PMC2823603

[16] Beutel ME, Brähler E, Wiltink J, et al. Emotional and tangible social support in a German population-based sample: Development and validation of the Brief Social Support Scale (BS6). PloS One. 2017;12(10):e0186516. DOI: 10.1371/journal.pone.0186516 29023540 PMC5638535

[17] Hair JF, Black WC, Babin BJ, Anderson RE. Multivariate Data Analysis. 7th ed. Pearson; 2014. Accessed January 15, 2019. https://www.pearson.com/us/higher-education/program/Hair-Multivariate-Data-Analysis-7th-Edition/PGM263675.html

[18] Raykov T, Marcoulides GA. A First Course in Structural Equation Modeling, 2nd Ed. Lawrence Erlbaum Associates Publishers; 2006:ix, 238.

[19] Cunningham CT, Quan H, Hemmelgarn B, et al. Exploring physician specialist response rates to web-based surveys. BMC Med Res Methodol. 2015;15:32. DOI: 10.1186/s12874-015-0016-z 25888346 PMC4404667

[20] Cook JV, Dickinson HO, Eccles MP. Response rates in postal surveys of healthcare professionals between 1996 and 2005: An observational study. BMC Health Serv Res. 2009;9(1):160. DOI: 10.1186/1472-6963-9-160 19751504 PMC2758861

[21] Lu DW, Zhan T, Bilimoria KY, et al. Workplace Mistreatment, Career Choice Regret, and Burnout in Emergency Medicine Residency Training in the United States. Ann Emerg Med. 2023;81(6):706–714. DOI: 10.1016/j.annemergmed.2022.10.015 36754699

[22] Fincham FD, Cain KM. Learned helplessness in humans: A developmental analysis. Dev Rev. 1986;6(4):301–333. DOI: 10.1016/0273-2297(86)90016-X

[23] Wang X, Wang H. How to survive mistreatment by customers: Employees’ work withdrawal and their coping resources. Int J Confl Manag. 2017;28(4):464–482. DOI: 10.1108/IJCMA-11-2016-0089

[24] Wolf TM, Scurria PL, Webster MG. A Four-year Study of Anxiety, Depression, Loneliness, Social Support, and Perceived Mistreatment in Medical Students. J Health Psychol. 1998;3(1):125–136. DOI: 10.1177/135910539800300110 22021348

[25] Yao J, Lim S, Guo CY, Ou AY, Ng JWX. Experienced incivility in the workplace: A meta-analytical review of its construct validity and nomological network. J Appl Psychol. 2022;107(2):193–220. DOI: 10.1037/apl0000870 33914571

[26] Laschinger HKS, Cummings G, Leiter M, et al. Starting Out: A time-lagged study of new graduate nurses’ transition to practice. Int J Nurs Stud. 2016;57:82–95. DOI: 10.1016/j.ijnurstu.2016.01.005 27045567

[27] Cohen S, Wills TA. Stress, social support, and the buffering hypothesis. Psychol Bull. 1985;98(2):310–357. DOI: 10.1037/0033-2909.98.2.310 3901065

[28] Halbesleben JRB. Sources of social support and burnout: a meta-analytic test of the conservation of resources model. J Appl Psychol. 2006;91(5):1134–1145. DOI: 10.1037/0021-9010.91.5.1134 16953774

[29] Brazeau CMLR, Shanafelt T, Durning SJ, et al. Distress among matriculating medical students relative to the general population. Acad Med J Assoc Am Med Coll. 2014;89(11):1520–1525. DOI: 10.1097/ACM.0000000000000482 25250752

[30] Lockspeiser TM, O’Sullivan P, Teherani A, Muller J. Understanding the experience of being taught by peers: the value of social and cognitive congruence. Adv Health Sci Educ Theory Pract. 2008;13(3):361–372. DOI: 10.1007/s10459-006-9049-8 17124627

[31] Versteeg M, van Blankenstein FM, Putter H, Steendijk P. Peer instruction improves comprehension and transfer of physiological concepts: a randomized comparison with self-explanation. Adv Health Sci Educ. 2019;24(1):151–165. DOI: 10.1007/s10459-018-9858-6 PMC6373526 30343408

[32] Edmondson A. Psychological safety and learning behavior in work teams. Adm Sci Q. 1999;44(2):350–383. DOI: 10.2307/2666999

[33] West CP, Dyrbye LN, Erwin PJ, Shanafelt TD. Interventions to Prevent and Reduce Physician Burnout: A Systematic Review and Meta-Analysis. Lancet. 2016;388(10057):2272–2281. DOI: 10.1016/S0140-6736(16)31279-X 27692469

[34] Doulougeri K, Panagopoulou E, Montgomery A. (How) do medical students regulate their emotions? BMC Med Educ. 2016;16(1):312. DOI: 10.1186/s12909-016-0832-9 27955653 PMC5154027

[35] Merkebu J, Veen M, Hosseini S, Varpio L. The case for metacognitive reflection: a theory integrative review with implications for medical education. Adv Health Sci Educ. 2024;29(4):1481–1500. DOI: 10.1007/s10459-023-10310-2 PMC11368986 38345690

[36] Gan R, Snell L. When the learning environment is suboptimal: exploring medical students’ perceptions of “mistreatment.” Acad Med J Assoc Am Med Coll. 2014;89(4):608–617. DOI: 10.1097/ACM.0000000000000172 PMC4885564 24556767

